# Diethyl [(4-nitrobenzamido)(phen­yl)meth­yl]phospho­nate

**DOI:** 10.1107/S1600536814007776

**Published:** 2014-04-12

**Authors:** Jing-Wei Chen, Bai-Cun Li, Hua Fang, Zhen Wu, Mei-Juan Fang

**Affiliations:** a The Key Laboratory for Chemical Biology of Fujian Province, Xiamen University, Xiamen 361005, People’s Republic of China; b School of Pharmaceutical Sciences, Xiamen University, Xiang-An South Road, Xiamen 361100, People’s Republic of China; c The Third Institute of Oceanography of the State Oceanic Administration, Xiamen 361005, People’s Republic of China

## Abstract

In the title compound, C_18_H_21_N_2_O_6_P, the dihedral angle between the benzene and phenyl rings is 85.1 (2)°. In the crystal, mol­ecules are linked *via* pairs of N—H⋯O(=P) hydrogen bonds, forming inversion dimers with graph-set notation *R*_2_^2^(10). One of the ethyl groups is disordered over two sets of sites, with occupancies 0.746 (11) and 0.254 (11).

## Related literature   

For the synthesis, see: Takahashi *et al.* (1994 ▶). For a related structure, see: Fang *et al.* (2004 ▶). For hydrogen bond graph-set notation, see: Bernstein *et al.* (1995 ▶).
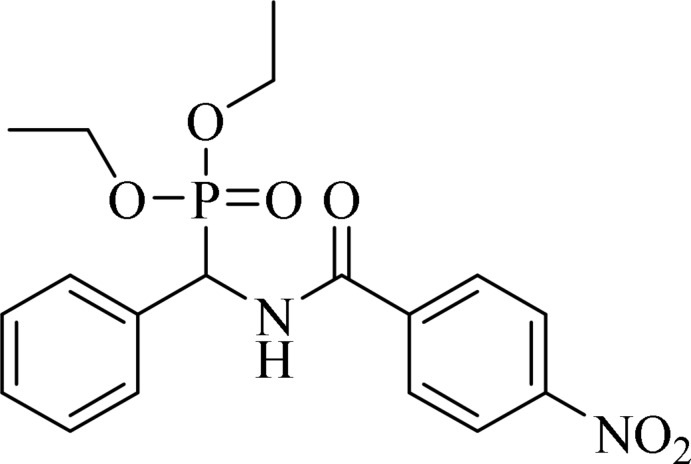


## Experimental   

### 

#### Crystal data  

C_18_H_21_N_2_O_6_P
*M* *_r_* = 392.34
Triclinic, 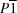

*a* = 8.112 (3) Å
*b* = 10.378 (4) Å
*c* = 12.583 (5) Å
α = 106.321 (7)°
β = 90.188 (8)°
γ = 106.035 (7)°
*V* = 973.3 (6) Å^3^
*Z* = 2
Mo *K*α radiation
μ = 0.18 mm^−1^
*T* = 293 K
0.42 × 0.28 × 0.23 mm

#### Data collection  

Bruker APEX diffractometer
Absorption correction: multi-scan (*SADABS*; Bruker, 2001 ▶) *T*_min_ = 0.929, *T*_max_ = 0.960
4948 measured reflections
3375 independent reflections
2719 reflections with *I* > 2σ(*I*)
*R*_int_ = 0.028

#### Refinement  

*R*[*F* ^2^ > 2σ(*F* ^2^)] = 0.075
*wR*(*F* ^2^) = 0.200
*S* = 1.09
3375 reflections
259 parameters
1 restraint
H atoms treated by a mixture of independent and constrained refinement
Δρ_max_ = 0.48 e Å^−3^
Δρ_min_ = −0.23 e Å^−3^

### 

Data collection: *SMART* (Bruker, 2001 ▶); cell refinement: *SAINT* (Bruker, 2001 ▶); data reduction: *SAINT*; program(s) used to solve structure: *SHELXS97* (Sheldrick, 2008 ▶); program(s) used to refine structure: *SHELXL97* (Sheldrick, 2008 ▶); molecular graphics: *ORTEP-3 for Windows* (Farrugia, 2012 ▶); software used to prepare material for publication: *SHELXL97*.

## Supplementary Material

Crystal structure: contains datablock(s) I. DOI: 10.1107/S1600536814007776/lh5697sup1.cif

Structure factors: contains datablock(s) I. DOI: 10.1107/S1600536814007776/lh5697Isup2.hkl

Supporting information file. DOI: 10.1107/S1600536814007776/lh5697Isup3.cml

CCDC reference: 995975

Additional supporting information:  crystallographic information; 3D view; checkCIF report

## Figures and Tables

**Table 1:** Hydrogen-bond geometry (Å, °)

*D*—H⋯*A*	*D*—H	H⋯*A*	*D*⋯*A*	*D*—H⋯*A*
N1—H1*N*⋯O2^i^	0.78 (4)	2.15 (4)	2.909 (4)	164 (4)

Symmetry code: (i) 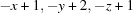.

**Fig. 1.**
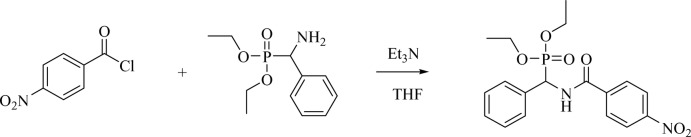
The reaction scheme.

**Fig. 2.**
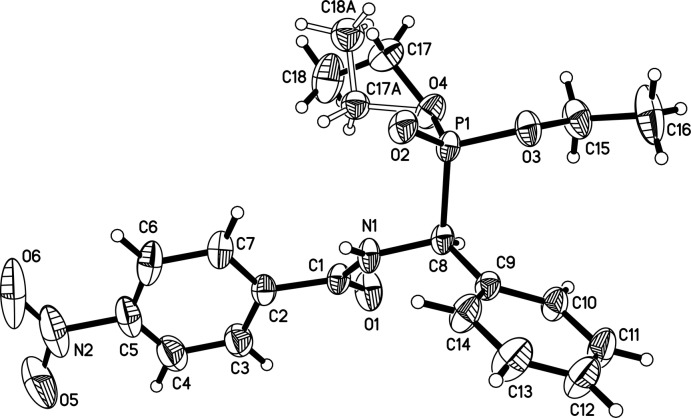
The molecular structure of the title compound with displacement ellipsoids drawn at the 30% probability level. H atoms are drawn as spheres of arbitrary radii and the open bonds indicate the minor component of disorder.

## supplementary crystallographic information

### 1. Comment

The title compound (I) was synthesized for a study of its antimicrobial activity against Bacillus subtilis. This aminophosphonate derivative was found to have weak antimicrobial activity (inhibition zone = 7 mm). The molecular structure of the title compound is shown in Fig. 1. The dihedral angle between the benzene (C2–C7) ring and phenyl (C9–C14) ring is 85.1 (2)°. In the crystal, molecules are linked *via* pairs of N—H···O(═P) hydrogen bonds forming inversion dimers with with graph-set notation *R*^2^_2_(10) (Bernstein *et al.*, 1995). One of the ethyl groups (C17/C18) is disordered over two sets of sites with occupancies 0.746 (11) and 0.254 (11). Bond lenths and angles in (I) are in agreement with the values reported for a similar structure (Fang *et al.*, 2004).

### 2. Experimental

The hydrochloride of diethyl amino(phenyl)methylphosphonate was prepared according to the literature procedure (Takahashi *et al.*, 1994). This ester (1.08 g, 5 mmol) was dissolved in dry tetrahydrofuran (20 ml) to which triethylamine (0.7 ml) was added, and the solution was added dropwise to 4-nitrobenzoyl chloride (0.9 g, 5 mmol) in the same solvent (10 ml) (see Fig. 1). After completion of the reaction, the precipitate was separated and the filtrate was extracted with ethyl acetate, dried over anhydrous MgSO_4_ and concentrated under vacuum. The residual liquid was purified by column chromatography to give the title compound. Single crystals suitable for X-ray analysis were obtained by slow evaporation of a petroleum ether - ethyl acetate solution (3:1 *v*/*v*) of the title compound.

### 3. Refinement

The H atoms were positioned geometrically (C—H = 0.93–0.98 Å) and were included in the refinement in the riding-model approximation with *U*_iso_(H) = 1.2*U*_eq_(C) or 1.5*U*_eq_(C_methyl_). The H atom bonded to the N atom was refined independently with *U*_iso_(H) = 1.2*U*_eq_(N).

### Crystal data

**Table:**

C_18_H_21_N_2_O_6_P	*Z* = 2
*M**_r_* = 392.34	*F*(000) = 412
Triclinic, *P*1	*D*_x_ = 1.339 Mg m^−^^3^
Hall symbol: -P 1	Mo *K*α radiation, λ = 0.71073 Å
*a* = 8.112 (3) Å	Cell parameters from 1770 reflections
*b* = 10.378 (4) Å	θ = 1.3–26.6°
*c* = 12.583 (5) Å	µ = 0.18 mm^−^^1^
α = 106.321 (7)°	*T* = 293 K
β = 90.188 (8)°	Block, colorless
γ = 106.035 (7)°	0.42 × 0.28 × 0.23 mm
*V* = 973.3 (6) Å^3^	

### Data collection

**Table:**

Bruker APEX diffractometer	3375 independent reflections
Radiation source: fine-focus sealed tube	2719 reflections with *I* > 2σ(*I*)
Graphite monochromator	*R*_int_ = 0.028
φ and ω scan	θ_max_ = 25.0°, θ_min_ = 2.1°
Absorption correction: multi-scan (*SADABS*; Bruker, 2001)	*h* = −9→9
*T*_min_ = 0.929, *T*_max_ = 0.960	*k* = −12→12
4948 measured reflections	*l* = −8→14

### Refinement

**Table:**

Refinement on *F*^2^	Primary atom site location: structure-invariant direct methods
Least-squares matrix: full	Secondary atom site location: difference Fourier map
*R*[*F*^2^ > 2σ(*F*^2^)] = 0.075	Hydrogen site location: inferred from neighbouring sites
*wR*(*F*^2^) = 0.200	H atoms treated by a mixture of independent and constrained refinement
*S* = 1.09	*w* = 1/[σ^2^(*F*_o_^2^) + (0.1021*P*)^2^ + 0.2844*P*] where *P* = (*F*_o_^2^ + 2*F*_c_^2^)/3
3375 reflections	(Δ/σ)_max_ = 0.013
259 parameters	Δρ_max_ = 0.48 e Å^−^^3^
1 restraint	Δρ_min_ = −0.23 e Å^−^^3^

### Special details

**Table:**

Geometry. All e.s.d.'s (except the e.s.d. in the dihedral angle between two l.s. planes) are estimated using the full covariance matrix. The cell e.s.d.'s are taken into account individually in the estimation of e.s.d.'s in distances, angles and torsion angles; correlations between e.s.d.'s in cell parameters are only used when they are defined by crystal symmetry. An approximate (isotropic) treatment of cell e.s.d.'s is used for estimating e.s.d.'s involving l.s. planes.
Refinement. Refinement of *F*^2^ against ALL reflections. The weighted *R*-factor *wR* and goodness of fit *S* are based on *F*^2^, conventional *R*-factors *R* are based on *F*, with *F* set to zero for negative *F*^2^. The threshold expression of *F*^2^ > σ(*F*^2^) is used only for calculating *R*-factors(gt) *etc*. and is not relevant to the choice of reflections for refinement. *R*-factors based on *F*^2^ are statistically about twice as large as those based on *F*, and *R*- factors based on ALL data will be even larger.

### Fractional atomic coordinates and isotropic or equivalent isotropic displacement parameters (Å^2^)

**Table:**

	*x*	*y*	*z*	*U*_iso_*/*U*_eq_	Occ. (<1)
P1	0.32681 (11)	0.73988 (8)	0.39408 (7)	0.0472 (3)	
O1	0.5803 (4)	0.6502 (2)	0.6389 (2)	0.0749 (8)	
O2	0.3346 (3)	0.8870 (2)	0.41776 (19)	0.0576 (6)	
O3	0.2957 (3)	0.6646 (2)	0.26662 (18)	0.0591 (7)	
O4	0.1843 (3)	0.6511 (3)	0.4480 (2)	0.0675 (7)	
O5	0.8608 (8)	1.1843 (7)	1.1263 (4)	0.195 (3)	
O6	0.6792 (9)	1.2811 (5)	1.0861 (4)	0.172 (3)	
N1	0.5688 (4)	0.8136 (3)	0.5566 (2)	0.0510 (8)	
H1N	0.576 (5)	0.893 (4)	0.567 (3)	0.061*	
N2	0.7490 (9)	1.1926 (6)	1.0645 (4)	0.124 (2)	
C1	0.5887 (4)	0.7714 (3)	0.6449 (3)	0.0504 (8)	
C2	0.6243 (4)	0.8826 (3)	0.7554 (3)	0.0513 (8)	
C3	0.7279 (5)	0.8703 (5)	0.8364 (3)	0.0724 (11)	
H3A	0.7716	0.7938	0.8219	0.087*	
C4	0.7674 (6)	0.9689 (6)	0.9376 (4)	0.0849 (13)	
H4A	0.8371	0.9599	0.9923	0.102*	
C5	0.7041 (6)	1.0796 (5)	0.9574 (3)	0.0797 (14)	
C6	0.5969 (6)	1.0951 (4)	0.8798 (3)	0.0790 (13)	
H6A	0.5523	1.1712	0.8957	0.095*	
C7	0.5574 (5)	0.9937 (4)	0.7773 (3)	0.0633 (10)	
H7A	0.4852	1.0014	0.7232	0.076*	
C8	0.5261 (4)	0.7180 (3)	0.4444 (2)	0.0469 (8)	
H8A	0.5015	0.6220	0.4493	0.056*	
C9	0.6651 (4)	0.7392 (3)	0.3678 (3)	0.0470 (8)	
C10	0.6906 (5)	0.6247 (4)	0.2896 (3)	0.0609 (9)	
H10A	0.6227	0.5351	0.2863	0.073*	
C11	0.8151 (6)	0.6411 (5)	0.2164 (4)	0.0807 (13)	
H11A	0.8304	0.5630	0.1638	0.097*	
C12	0.9163 (6)	0.7726 (5)	0.2213 (4)	0.0899 (14)	
H12A	1.0009	0.7845	0.1722	0.108*	
C13	0.8918 (6)	0.8858 (5)	0.2989 (4)	0.0892 (14)	
H13A	0.9605	0.9752	0.3024	0.107*	
C14	0.7690 (5)	0.8704 (4)	0.3711 (3)	0.0676 (10)	
H14A	0.7548	0.9492	0.4234	0.081*	
C15	0.2262 (6)	0.5160 (4)	0.2178 (3)	0.0771 (12)	
H15A	0.1059	0.4863	0.2312	0.092*	
H15B	0.2881	0.4670	0.2508	0.092*	
C16	0.2434 (10)	0.4827 (5)	0.0984 (4)	0.127 (2)	
H16A	0.1971	0.3834	0.0647	0.191*	
H16B	0.3628	0.5116	0.0857	0.191*	
H16C	0.1814	0.5312	0.0663	0.191*	
C17	0.0434 (8)	0.6943 (7)	0.5001 (5)	0.077 (2)	0.746 (11)
H17A	0.0383	0.7802	0.4856	0.092*	0.746 (11)
H17B	−0.0641	0.6225	0.4697	0.092*	0.746 (11)
C18	0.0671 (14)	0.7168 (8)	0.6179 (5)	0.114 (3)	0.746 (11)
H18A	−0.0335	0.7344	0.6517	0.171*	0.746 (11)
H18B	0.1655	0.7961	0.6491	0.171*	0.746 (11)
H18C	0.0851	0.6350	0.6314	0.171*	0.746 (11)
C17A	0.1513 (16)	0.7129 (17)	0.5723 (15)	0.060 (5)*	0.254 (11)
H17C	0.1723	0.6563	0.6175	0.072*	0.254 (11)
H17D	0.2236	0.8086	0.6033	0.072*	0.254 (11)
C18A	−0.0275 (17)	0.707 (2)	0.565 (2)	0.082 (7)*	0.254 (11)
H18D	−0.0526	0.7677	0.6316	0.123*	0.254 (11)
H18E	−0.0973	0.6127	0.5546	0.123*	0.254 (11)
H18F	−0.0521	0.7373	0.5023	0.123*	0.254 (11)

### Atomic displacement parameters (Å^2^)

**Table:**

	*U*^11^	*U*^22^	*U*^33^	*U*^12^	*U*^13^	*U*^23^
P1	0.0631 (6)	0.0300 (5)	0.0477 (5)	0.0161 (4)	0.0095 (4)	0.0075 (3)
O1	0.130 (2)	0.0410 (14)	0.0592 (15)	0.0318 (15)	0.0001 (15)	0.0164 (12)
O2	0.0741 (15)	0.0361 (12)	0.0650 (15)	0.0244 (11)	0.0064 (12)	0.0104 (11)
O3	0.0810 (17)	0.0402 (13)	0.0496 (14)	0.0137 (11)	0.0008 (12)	0.0066 (10)
O4	0.0772 (17)	0.0490 (15)	0.0789 (18)	0.0217 (13)	0.0268 (14)	0.0190 (13)
O5	0.184 (5)	0.208 (6)	0.104 (3)	0.025 (4)	−0.043 (4)	−0.058 (4)
O6	0.290 (8)	0.074 (3)	0.094 (3)	0.009 (4)	0.056 (4)	−0.024 (2)
N1	0.085 (2)	0.0280 (13)	0.0423 (15)	0.0223 (14)	0.0062 (13)	0.0075 (12)
N2	0.149 (5)	0.095 (4)	0.063 (3)	−0.031 (3)	0.013 (3)	−0.013 (3)
C1	0.069 (2)	0.0373 (17)	0.0475 (19)	0.0213 (15)	0.0079 (16)	0.0103 (14)
C2	0.063 (2)	0.0424 (18)	0.0457 (18)	0.0104 (16)	0.0098 (16)	0.0135 (15)
C3	0.083 (3)	0.074 (3)	0.057 (2)	0.026 (2)	−0.002 (2)	0.012 (2)
C4	0.089 (3)	0.091 (3)	0.058 (2)	0.012 (3)	−0.007 (2)	0.008 (2)
C5	0.092 (3)	0.067 (3)	0.045 (2)	−0.019 (2)	0.016 (2)	0.0007 (19)
C6	0.119 (4)	0.043 (2)	0.065 (3)	0.015 (2)	0.037 (3)	0.0082 (18)
C7	0.093 (3)	0.048 (2)	0.049 (2)	0.0206 (19)	0.0153 (19)	0.0135 (16)
C8	0.072 (2)	0.0264 (15)	0.0436 (17)	0.0201 (15)	0.0071 (15)	0.0066 (13)
C9	0.0592 (19)	0.0405 (17)	0.0454 (17)	0.0242 (15)	0.0042 (15)	0.0093 (14)
C10	0.073 (2)	0.046 (2)	0.058 (2)	0.0202 (17)	0.0117 (18)	0.0023 (16)
C11	0.097 (3)	0.065 (3)	0.071 (3)	0.031 (2)	0.027 (2)	−0.001 (2)
C12	0.090 (3)	0.087 (3)	0.093 (3)	0.032 (3)	0.045 (3)	0.021 (3)
C13	0.094 (3)	0.060 (3)	0.115 (4)	0.024 (2)	0.045 (3)	0.026 (3)
C14	0.083 (3)	0.0388 (19)	0.082 (3)	0.0241 (18)	0.029 (2)	0.0121 (18)
C15	0.112 (3)	0.041 (2)	0.068 (3)	0.021 (2)	−0.009 (2)	0.0017 (18)
C16	0.230 (7)	0.070 (3)	0.058 (3)	0.031 (4)	−0.006 (4)	−0.007 (2)
C17	0.072 (4)	0.077 (4)	0.093 (5)	0.030 (3)	0.024 (4)	0.033 (3)
C18	0.160 (9)	0.101 (6)	0.080 (5)	0.033 (5)	0.055 (6)	0.028 (4)

### Geometric parameters (Å, º)

**Table:**

P1—O2	1.454 (2)	C9—C10	1.379 (5)
P1—O4	1.553 (3)	C10—C11	1.377 (5)
P1—O3	1.559 (2)	C10—H10A	0.9300
P1—C8	1.829 (3)	C11—C12	1.369 (6)
O1—C1	1.222 (4)	C11—H11A	0.9300
O3—C15	1.435 (4)	C12—C13	1.364 (6)
O4—C17	1.435 (6)	C12—H12A	0.9300
O4—C17A	1.569 (18)	C13—C14	1.358 (6)
O5—N2	1.229 (9)	C13—H13A	0.9300
O6—N2	1.178 (9)	C14—H14A	0.9300
N1—C1	1.328 (4)	C15—C16	1.460 (6)
N1—C8	1.454 (4)	C15—H15A	0.9700
N1—H1N	0.78 (4)	C15—H15B	0.9700
N2—C5	1.482 (6)	C16—H16A	0.9600
C1—C2	1.503 (5)	C16—H16B	0.9600
C2—C7	1.367 (5)	C16—H16C	0.9600
C2—C3	1.374 (5)	C17—C18	1.437 (8)
C3—C4	1.363 (6)	C17—H17A	0.9700
C3—H3A	0.9300	C17—H17B	0.9700
C4—C5	1.345 (7)	C18—H18A	0.9600
C4—H4A	0.9300	C18—H18B	0.9600
C5—C6	1.377 (7)	C18—H18C	0.9600
C6—C7	1.387 (5)	C17A—C18A	1.437 (8)
C6—H6A	0.9300	C17A—H17C	0.9700
C7—H7A	0.9300	C17A—H17D	0.9700
C8—C9	1.497 (5)	C18A—H18D	0.9600
C8—H8A	0.9800	C18A—H18E	0.9600
C9—C14	1.379 (5)	C18A—H18F	0.9600
			
O2—P1—O4	115.90 (15)	C11—C10—C9	121.0 (4)
O2—P1—O3	111.00 (13)	C11—C10—H10A	119.5
O4—P1—O3	105.86 (14)	C9—C10—H10A	119.5
O2—P1—C8	111.80 (13)	C12—C11—C10	119.8 (4)
O4—P1—C8	103.95 (15)	C12—C11—H11A	120.1
O3—P1—C8	107.74 (14)	C10—C11—H11A	120.1
C15—O3—P1	124.6 (2)	C13—C12—C11	119.2 (4)
C17—O4—P1	125.5 (3)	C13—C12—H12A	120.4
C17—O4—C17A	46.7 (5)	C11—C12—H12A	120.4
P1—O4—C17A	119.6 (6)	C14—C13—C12	121.3 (4)
C1—N1—C8	122.9 (3)	C14—C13—H13A	119.4
C1—N1—H1N	118 (3)	C12—C13—H13A	119.4
C8—N1—H1N	119 (3)	C13—C14—C9	120.7 (4)
O6—N2—O5	124.5 (5)	C13—C14—H14A	119.7
O6—N2—C5	120.8 (7)	C9—C14—H14A	119.7
O5—N2—C5	114.8 (7)	O3—C15—C16	108.7 (4)
O1—C1—N1	123.2 (3)	O3—C15—H15A	110.0
O1—C1—C2	120.7 (3)	C16—C15—H15A	110.0
N1—C1—C2	116.1 (3)	O3—C15—H15B	110.0
C7—C2—C3	119.6 (3)	C16—C15—H15B	110.0
C7—C2—C1	122.4 (3)	H15A—C15—H15B	108.3
C3—C2—C1	118.0 (3)	C15—C16—H16A	109.5
C4—C3—C2	120.8 (4)	C15—C16—H16B	109.5
C4—C3—H3A	119.6	H16A—C16—H16B	109.5
C2—C3—H3A	119.6	C15—C16—H16C	109.5
C5—C4—C3	119.1 (4)	H16A—C16—H16C	109.5
C5—C4—H4A	120.5	H16B—C16—H16C	109.5
C3—C4—H4A	120.5	C18—C17—O4	109.3 (6)
C4—C5—C6	122.3 (4)	C18—C17—H17A	109.8
C4—C5—N2	121.2 (6)	O4—C17—H17A	109.8
C6—C5—N2	116.5 (6)	C18—C17—H17B	109.8
C5—C6—C7	118.0 (4)	O4—C17—H17B	109.8
C5—C6—H6A	121.0	H17A—C17—H17B	108.3
C7—C6—H6A	121.0	C18A—C17A—O4	102.9 (15)
C2—C7—C6	120.2 (4)	C18A—C17A—H17C	111.2
C2—C7—H7A	119.9	O4—C17A—H17C	111.2
C6—C7—H7A	119.9	C18A—C17A—H17D	111.2
N1—C8—C9	114.6 (3)	O4—C17A—H17D	111.2
N1—C8—P1	105.6 (2)	H17C—C17A—H17D	109.1
C9—C8—P1	112.3 (2)	C17A—C18A—H18D	109.5
N1—C8—H8A	108.1	C17A—C18A—H18E	109.5
C9—C8—H8A	108.1	H18D—C18A—H18E	109.5
P1—C8—H8A	108.1	C17A—C18A—H18F	109.5
C14—C9—C10	118.0 (3)	H18D—C18A—H18F	109.5
C14—C9—C8	122.3 (3)	H18E—C18A—H18F	109.5
C10—C9—C8	119.7 (3)		
			
O2—P1—O3—C15	−158.4 (3)	C1—C2—C7—C6	−178.7 (3)
O4—P1—O3—C15	−31.9 (3)	C5—C6—C7—C2	−0.1 (6)
C8—P1—O3—C15	78.9 (3)	C1—N1—C8—C9	−113.4 (3)
O2—P1—O4—C17	12.4 (4)	C1—N1—C8—P1	122.6 (3)
O3—P1—O4—C17	−111.2 (4)	O2—P1—C8—N1	46.0 (2)
C8—P1—O4—C17	135.5 (4)	O4—P1—C8—N1	−79.8 (2)
O2—P1—O4—C17A	−43.3 (6)	O3—P1—C8—N1	168.18 (18)
O3—P1—O4—C17A	−166.8 (6)	O2—P1—C8—C9	−79.5 (2)
C8—P1—O4—C17A	79.8 (6)	O4—P1—C8—C9	154.8 (2)
C8—N1—C1—O1	3.3 (6)	O3—P1—C8—C9	42.7 (2)
C8—N1—C1—C2	−177.2 (3)	N1—C8—C9—C14	−37.0 (4)
O1—C1—C2—C7	−147.5 (4)	P1—C8—C9—C14	83.4 (4)
N1—C1—C2—C7	32.9 (5)	N1—C8—C9—C10	143.8 (3)
O1—C1—C2—C3	32.3 (5)	P1—C8—C9—C10	−95.8 (3)
N1—C1—C2—C3	−147.2 (3)	C14—C9—C10—C11	−0.5 (6)
C7—C2—C3—C4	−1.2 (6)	C8—C9—C10—C11	178.6 (3)
C1—C2—C3—C4	178.9 (4)	C9—C10—C11—C12	0.4 (7)
C2—C3—C4—C5	−0.3 (7)	C10—C11—C12—C13	−0.1 (8)
C3—C4—C5—C6	1.7 (7)	C11—C12—C13—C14	−0.1 (8)
C3—C4—C5—N2	−177.5 (4)	C12—C13—C14—C9	−0.1 (8)
O6—N2—C5—C4	−171.8 (5)	C10—C9—C14—C13	0.4 (6)
O5—N2—C5—C4	7.7 (7)	C8—C9—C14—C13	−178.8 (4)
O6—N2—C5—C6	9.0 (7)	P1—O3—C15—C16	−170.5 (4)
O5—N2—C5—C6	−171.6 (5)	P1—O4—C17—C18	−110.7 (5)
C4—C5—C6—C7	−1.5 (6)	C17A—O4—C17—C18	−11.4 (9)
N2—C5—C6—C7	177.7 (3)	C17—O4—C17A—C18A	10.2 (11)
C3—C2—C7—C6	1.4 (5)	P1—O4—C17A—C18A	122.7 (11)

### Hydrogen-bond geometry (Å, º)

**Table:**

*D*—H···*A*	*D*—H	H···*A*	*D*···*A*	*D*—H···*A*
N1—H1*N*···O2^i^	0.78 (4)	2.15 (4)	2.909 (4)	164 (4)

Symmetry code: (i) −*x*+1, −*y*+2, −*z*+1.
